# The Relationship between in-Vehicle Technologies and Self-Regulation among Older Drivers

**DOI:** 10.3390/geriatrics5020023

**Published:** 2020-04-16

**Authors:** Austin M. Svancara, Leon Villavicencio, Tara Kelley-Baker, William J. Horrey, Lisa J. Molnar, David W. Eby, Thelma J. Mielenz, Linda Hill, Carolyn DiGuiseppi, David Strogatz, Guohua Li

**Affiliations:** 1 University of Alabama at Birmingham, Birmingham, AL 35205, USA; 2 AAA Foundation for Traffic Safety, Washington, DC 20005, USA; lvillavicencio@aaafoundation.org (L.V.); tkelleybaker@aaafoundation.org (T.K.-B.); whorrey@aaafoundation.org (W.J.H.); 3 University of Michigan Transportation Research Institute, Ann Arbor, MI 48109, USA; ljmolnar@umich.edu (L.J.M.); eby@umich.edu (D.W.E.); 4 Columbia University Medical Center, New York, NY 10032, USA; tjm2141@cumc.columbia.edu; 5 San Diego Department of Family and Preventive Medicine, University of California San Diego, San Diego, CA 92093, USA; llhill@ucsd.edu; 6 University of Colorado Anschutz Medical Campus, Aurora, CO 80045, USA; carolyn.diguiseppi@cuanschutz.edu; 7 Bassett Research Institute, Bassett Healthcare Network, Cooperstown, NY 13326, USA; david.strogatz@bassett.org; 8 Department of Epidemiology, Mailman School of Public Health, and the Center for Injury Epidemiology and Prevention, Columbia University, New York, NY 10032, USA; gl2240@cumc.columbia.edu

**Keywords:** aging, transportation, driving behavior

## Abstract

The study sought to understand the relationship between in-vehicle technologies (IVTs) and self-regulatory behaviors among older drivers. In a large multi-site study of 2990 older drivers, self-reported data on the presence of IVTs and avoidance of various driving behaviors (talking on a mobile phone while driving, driving at night, driving in bad weather, and making left turns when there is no left turn arrow) were recorded. Self-reports were used to identify whether avoidance was due to self-regulation. Hierarchical logistic regressions were used to determine whether the presence of a particular IVT predicted the likelihood of a given self-regulatory behavior after controlling for other factors. Results suggest that the presence of Integrated Bluetooth/Voice Control systems are related to a reduced likelihood of avoiding talking on a mobile phone while driving due to self-regulation (OR = 0.37, 95% CI = 0.29–0.47). The presence of a Navigation Assistance system was related to a reduced likelihood of avoiding talking on a mobile phone while driving (OR= 0.65, 95% CI = 0.50–0.84) and avoiding driving at night due to self-regulation (OR = 0.80, 95% CI = 0.64–1.00). Present findings suggest in-vehicle technologies may differently influence the self-regulatory behaviors of older drivers.

## 1. Introduction

Declines in physical, perceptual, and cognitive performance are common in the process of aging, and these declines could affect one’s driving performance and safety. To compensate for these declines in performance and extend their safe mobility, older adults often self-regulate their driving behaviors [1,2]. Self-regulation is generally considered to be the process by which older drivers avoid particular driving behaviors and situations (e.g., driving at night, in bad weather, on the freeway) or reduce their overall driving in response to awareness of functional declines in one’s abilities [3,4] or general feelings of discomfort towards driving [2,5]. In this regard, self-regulation may provide older drivers the opportunity to maintain their mobility for an extended period of time, which is critical given the relationship between having access to transportation and maintaining quality of life [6]. 

While self-regulation may be one means of reducing crash risk while retaining mobility among older drivers [4], the use of in-vehicle technologies (IVTs) may offer an additional layer of safety. IVTs consist of both crash-mitigation and convenience features which are intended to lessen the burden of some driving related tasks. For instance, a forward collision warning system will monitor the environment and alert the driver when a front-facing collision is imminent. In this regard, part of the task of monitoring the driving environment is supported by the system. Another example of this is navigation assistance systems that directs the driver through a designated route, thus alleviating the driver’s burden of planning and adjusting one’s route. For this reason, IVTs may be considered to be a potential solution for older adults to maintain safe mobility.

Prior literature indicates that, while IVTs are largely meant to improve safety or alleviate some aspect of the driving task, drivers have been shown to alter their behavior due to the presence of navigation assistance systems [7], forward collision warning systems [8,9], lane departure warning systems [10], and adaptive cruise control systems [11,12]. The use of navigation assistance systems appear related to greater confidence in driving in unfamiliar areas among older drivers [13]. A field study including older drivers among its sample indicated that the use of a lane departure warning system is related to increased use of the turn signal and better maintenance of lane position [14]. While these studies suggest older drivers adapt their behavior when using IVTs, little is known how the presence of an IVT may interact with more intentional behavior changes (i.e., self-regulatory behaviors) used by older drivers.

There are three main forms of self-regulation: strategic, tactical, and life-goal [2]. Strategic self-regulation pertains to older adults’ decisions about certain driving situations before they embark on the trip (e.g., driving at night, making left turns when there are no turn arrows, or driving in bad weather). Tactical self-regulation refers to behaviors engaged in while on the road (e.g., making maneuvers in traffic, or engaging in secondary activities). Life-goal self-regulation encompasses life decisions, motives, and attitudes that indirectly impact one’s driving [15]. An example of a life-goal strategy is purchasing a newer vehicle in response to concerns about vehicle safety [16]. In this respect, the acquisition of an IVT may be considered a form of a life-goal self-regulatory behavior if the purchase is intended to mitigate performance declines.

Consequently, the presence of IVTs may indirectly impact tactical or strategic self-regulatory behaviors. The potential relationship between a given IVT and a given self-regulatory behavior will depend on the function of the particular IVT. For instance, safety-related systems, such as blind spot warning, lane departure warning, forward collision warning, and adaptive headlight systems may enhance the driver’s perception of safety and security [17]. Utilizing a driving simulator, Takada and Shimoyama [18] observed a reduced mental workload among drivers when the system was equipped with a collision warning system. While perhaps an optimistic view is that drivers will compensate for the reduced mental workload by allocating cognitive, physical or other resources to other driving tasks, there is concern that drivers will use the opportunity to engage in secondary tasks, such as talking on a mobile phone while driving.

This is similarly the case for convenience IVTs, such as navigation assistance, voice control, integrated Bluetooth, and adaptive cruise control systems. A navigation assistance system would logically alleviate the task of planning one’s route and reading street signs. Hence, a navigation assistance system may benefit older drivers’ strategic behaviors [13]. Likewise, it is possible older drivers may feel more confident in their ability to drive in other situations when utilizing a navigation assistance system, such as driving at night. Adaptive cruise control systems may alleviate the driver’s workload [19,20]. As another example of the potential influence of convenience features, many drivers tend to be more willing to use hands-free devices than hand-held for conversations while driving [21]. Despite evidence indicating hands-free mobile device conversations remain a driving risk [22], nearly 63% of older drivers with an integrated Bluetooth system perceive the system as making them safer [17]. In this regard, such drivers with an integrated Bluetooth system may be more inclined to use the system as a means for hands-free conversations. Thus, it seems likely that crash avoidance and convenience features alike may impact an older drivers self-regulatory driving behavior.

The current study aimed to elucidate the relationship between IVTs and self-regulatory behaviors. There is wide variety in the functional capacities of the currently available IVTs. It was hypothesized that the presence of blind spot warning, lane departure warning, forward collision warning, adaptive cruise control, navigation assistance, and integrated Bluetooth systems would be associated with a decreased avoidance due to self-regulation of talking on a mobile phone while driving. In other words, it is expected drivers without these technologies will be more inclined to avoid talking on a mobile phone while driving due to self-regulation than drivers with these technologies. All of which may stem from a perceived benefit of the technology being present. Navigation assistance and adaptive headlights systems were hypothesized to be associated with decreased avoidance of driving in bad weather due to self-regulation. The presence of blind spot warning, lane departure warning, forward collision warning, adaptive cruise control, and navigation assistance systems were hypothesized to be related to reduced avoidance of driving at night due to self-regulation. Further, the presence of a navigation assistance system was hypothesized to be related to less avoidance of making a left turn when there is no left turn arrow.

## 2. Materials and Methods

### 2.1. Participants and Data

The analysis utilized baseline data from the AAA Longitudinal Research on Aging Drivers (AAA LongROAD) study. AAA LongROAD is a multisite (Ann Arbor, Michigan; Baltimore, Maryland; Cooperstown, New York; Denver, Colorado; and San Diego, California) longitudinal cohort study designed to collect data on the medical, behavioral, environmental and vehicle technological factors influencing older adults’ driving behavior and safety.

The LongROAD study aims to answer a variety of questions on topics regarding older adults’ driving including: protective and risk factors for driving safety, the effect of medications on driving behavior and safety, self-regulation of driving, prevalence and perception of in-vehicle technologies and aftermarket adaptations, and the determinants and consequences of driving cessation.

Participants were 2990 active older drivers, aged 65 to 79 years at baseline, identified and recruited through screening of electronic medical records through primary care clinics affiliated with the study sites. Each of the five study sites aimed to recruit an approximately equal distribution across three age categories: 65–69, 70–74, and 75–79.

To ensure that the study participants were relatively healthy active drivers, inclusion criteria included: is between age 65 and 79 with a valid driver’s license, drives on average at least once a week; resides in the catchment areas for at least 10 months a year with no plans on moving out of the area within 5 years; drives a vehicle model year 1996 or newer at least 80% of the time; and has no significant cognitive impairment. Participants were excluded if they had cognitive impairment, as revealed by a diagnosis of dementia in the medical record or by the score on the Six Item Screener [23,24]. As this study uses baseline data, all of the participants were active drivers without cognitive impairment (detailed data on demographic characteristics of the participants are presented in Table 1).

### 2.2. Assessment Materials

#### 2.2.1. Self-Regulation and Demographic Information

The LongROAD study collects information on demographics, driving avoidance and self-regulatory behaviors through the Driving, Health and Functioning Questionnaire developed by the LongROAD research team. Self-regulatory behaviors of interest in this study included avoidance of: (1) talking on a mobile phone while driving, (2) making left hand turns at intersections where there are no left arrow signs, (3) driving at night, and (4) driving in bad weather.

These avoidance behaviors were considered to be self-regulatory behaviors if participants indicated that they avoided these driving situations because of functional declines and functional difficulties such as difficulties seeing during the day or night, remembering things, or concentrating on more than one thing at a time, or reduced physical performance (strength, flexibility, or general mobility). Driving avoidance was also deemed to be a self-regulatory behavior if participants indicated that they avoided the behavior because they no longer feel comfortable or safe driving in that situation.

#### 2.2.2. In-Vehicle Technologies

The LongROAD study collects information on the presence of IVTs through the Vehicle Technology Questionnaire (VTQ) developed by the LongROAD research team. The VTQ asks questions about whether the participant has certain IVTs, if they use them, how they learned to use them, and if the technologies make them feel like a safer driver. IVTs of interest are described in Table 2. Night vision and fatigue/drowsy driver alert enhancement was also included in the VTQ; however due to the small number of participants who owned these technologies (0.7% and 1.3%, respectively), they were excluded from the analyses.

As noted in Zanier and colleagues [25], participants may have confused voice control technologies with integrated Bluetooth technologies. This conclusion derived largely from discrepancies between the proportion of voice control systems found during an inspection of the older driver’s vehicles and the proportion of voice control systems self-reported by the older drivers. Voice control technologies are systems that allow a driver to operate vehicle systems through spoken commands, while integrated Bluetooth technology allows a driver to connect his or her mobile phone to the on-board computer. These two systems were combined in the following analyses, such that integrated Bluetooth/voice control (IB/VC) was marked as present if participants indicated that they had either or both of these technologies in their vehicle.

### 2.3. Plan of Analysis

#### 2.3.1. IVTs and Self-Regulatory Behaviors

An IVT was only explored in its association with a given self-regulatory behavior if the technology would function as the manufacturer intended in the given situation and if the technology would be relevant to the behavior. For instance, the sensors of a lane departure warning system would not function appropriately in bad weather and, hence, the association between the system and the self-regulatory behavior were not explored. It is recognized that some older adults may not fully understand these functional limitations of sensors in bad weather, however several studies have demonstrated that older adults are aware these limitations may exist [26,27]. As another example, an integrated Bluetooth system would function in a situation where a driver is making a left turn when there is no left turn arrow; however, the system would likely have no impact on the driver’s decision to make a left turn. A navigation assistance system, in contrast, would function when a driver is making a left turn and the system may encourage the driver to make left turns should the system direct the driver to make such a turn on their route.

#### 2.3.2. Bivariate Analyses

Prevalence of IVTs and avoidance behaviors due to self-regulation among participants were analyzed using descriptive analytic techniques. Unadjusted odds ratio estimates with 95% CI were used to describe the bivariate association between the presence of IVTs and avoidance of certain behaviors and situations due to self-regulation. A *p*-value of 0.05 was considered statistically significant.

#### 2.3.3. Hierarchical Logistic Regression Analyses

For each of the four self-regulatory behaviors examined as outcomes in this study, a separate hierarchical logistic regression was conducted to assess the relationship between IVTs and the specified self-regulatory behavior [28,29]. Hierarchical logistic regressions were conducted as two models predicting the main outcome avoidance due to self-regulation. The first (reduced) model regressed only factors known to influence the described self-regulatory behaviors. Such factors were age, gender, race, income, and social factors [30,31,32,33]. Social factors included marital status, whether someone depended on the older driver for rides and whether the older driver had other people available to drive them. As data for this study were collected at multiple sites, a variable indicating the site of the data acquisition was also included in the first (Reduced) model to account for potential geographical factors. These predictors were added simultaneously to the model.

The second (full) model of the hierarchical procedure additionally included variables indicating the presence or absence of each relevant IVT. Such variables were added simultaneously to the model. An IVT was only included in the second model if in the prior bivariate analysis their association was statistically significant. Each IVT predictor was added with the referent being the absence of the specified technology. Likelihood ratio tests were used to determine whether the addition of IVTs improved the overall model.

## 3. Results

### 3.1. Descriptive: Prevalence of IVTs

Among IVTs analyzed in the entire sample at baseline, having IB/VC system (48.1%, n= 1438) was the most common, followed by navigation assistance system at 27.7% (n = 832), and blind spot warning system at 10.1% (n = 303). On the other hand, adaptive headlights at 3.6% (n = 109), and lane departure warning at 5.6% (n = 169) were the least prevalent technologies reported in this cohort. The prevalence and use of IVTs in the sample have been detailed previously [17].

### 3.2. Descriptive: Self-Regulatory Behaviors

Table 3 displays the frequency of self-regulatory behaviors (avoidance of driving while talking on a mobile phone, driving at night, making a left turn when there is no left turn arrow, and driving in bad weather) for the total sample and for drivers who had a specific IVT. The frequencies reported are the percentage of individuals who do not avoid these driving behaviors and of those who do avoid them due to self-regulation. Individuals who avoid a behavior for reasons other than self-regulation were removed from further analyses. Individuals who do not avoid and individuals who avoid for reasons other than self-regulation were not collapsed as there were demographics differences between the two groups. As illustrated in Table 3, the majority of participants in the total sample avoid talking on a mobile phone while driving due to self-regulation (53.3%). In contrast, few of the participants avoid making left turns when there are no left turn arrows due to self-regulation (10.4%).

### 3.3. Bivariate Analyses

Unadjusted odds ratios with 95% confidence intervals were calculated to determine if older drivers with specific IVTs have lower odds of self-regulating through the avoidance of certain driving situations and behaviors. As seen in Table 4, nearly all examined technologies were related to the relevant self-regulatory behavior.

### 3.4. Logistic Regression Analyses

The simultaneous addition of the IVT predictors (Block 2 of each model) improved the model for avoidance of talking on the phone while driving, χ^2^(6) = 338.95, *p* < 0.001. Within this particular model, age, gender, race, income, marital status, and others depending on the older driver for rides significantly predicted the likelihood of an older driver avoiding talking on a mobile phone while driving. The presence of IB/VC and navigation assistance systems were also found to be significant predictors. Specifically, drivers with these systems were less likely to avoid talking on the phone while driving due to self-regulation. Likewise, drivers with a navigation assistance system were less likely to avoid driving at night due to self-regulation than drivers without the system. The addition of the IVT predictors improved the model fit for the avoidance of driving at night, χ^2^(2) = 242.26, *p* < 0.05. While other IVTs were not significant predictors in the other two models, their addition to the models improved the model fit for the avoidance of driving in bad weather, χ^2^(2) = 183.57, *p* < 0.001, and the avoidance of left turns when there is no left turn arrow, χ^2^(1) = 23.55, *p* < 0.05. Across all of the models it seems with each older age group, participants were more likely to self-regulate talking on the phone while driving, driving at night, and making left turns when there is no left turn arrow. Older drivers who had an annual income of USD 100,000 or more were less likely to avoid talking on a mobile phone while driving, avoid driving in bad weather and avoid driving at night. Likewise, compared to males, females were more likely to avoid all driving situations due to self-regulation. The coefficients of the second (full) model for each self-regulatory behavior are displayed in Table 5 and Table 6. Results of the reduced model (i.e., age, gender, race, income, social factors, site location) are displayed in Table 5 and Table 6. The coefficients of the second (full) model for each self-regulatory behavior are displayed in Table 5. Results of the full model (e.g., each of the IVTs) are displayed in Table 6.

## 4. Discussion

### 4.1. In-Vehicle Technologies

The aim of the study was to investigate if IVTs were associated with driving self-regulation among older drivers. While we found that most IVTs analyzed in this study were not associated with self-regulatory behaviors, some convenience technologies such as IB/VC and navigation assistance systems were. In particular, older drivers with IB/VC and navigation technologies appeared less likely to avoid mobile phone conversations while driving. In this regard, the presence of both a navigation assistance and IB/VC system seems to hinder the safety of older drivers, in that these drivers appear more willing to engage in this distracting behavior than drivers without the system. Further, avoidance of driving at night due to self-regulation was less likely for participants with a navigation assistance system in their vehicle. In this case, older drivers may benefit from the presence of navigation assistance, as drivers with these systems appear more willing to drive at night and, as a result, maintain their mobility option to a greater extent than drivers without the technology.

There are tradeoffs to foregoing avoidance of self-regulatory behaviors due to the presence of these technologies. A false sense of security and comfort may put older drivers at risk as they may split their attentional resources in situations where they should be alert and vigilant. The finding that the presence of an IB/VC system is related to less avoidance of talking on a mobile phone appears to corroborate this potential risk. Drivers tend to perceive hands-free conversations as less risky than hand-held conversations [34]. Similarly, older drivers may perceive the use of an IB/VC as a safe alternative to the traditional handling of a mobile phone for conversing while driving. However, the IB/VC system does not address the cognitive demand of conversing on a mobile phone while driving. A meta-analysis by Caird and colleagues [22] indicated that there are still driving safety risks associated with hands-free phone conversations.

Similarly, while the use of navigation assistance systems may alleviate the stress of navigating the road, these systems also impose demands on visual attention [35]. Thus, because of the perceived safety benefits of the system, older drivers may also feel they can engage in secondary tasks. Present findings indicate that older drivers may be less likely to avoid talking on a mobile phone while driving when a navigation assistance system is present. A prior study exploring the same sample used in this study indicated that 62.4% of older drivers with a navigation assistance system perceived the system as making them safer [36]. Furthermore, the presence of a navigation assistance system is seemingly related to a lower likelihood of avoiding driving at night. It may be that such systems alleviate the task of searching and navigating the roadway at night, a task that likely becomes more difficult as the visibility of the road and signs declines. Of course, the presence of a navigation assistance system does not address the cognitive or visual function needed for detecting hazards on the road at night. These relationships need further investigating.

It was surprising to note that other IVTs did not show a statistically significant association with self-regulatory behaviors. The more advanced technologies, particularly the crash avoidance systems, are promising technologies that could assist drivers in driving tasks that could potentially free up attentional resources. It may be that the older drivers in this sample are not over-reliant on such systems. It could also be that purchasing a new vehicle (one with advanced vehicle technologies) may not be a typical driver’s response to declining health functioning. The process of life-goal level self-regulation, which has to do with the larger life decisions such as purchasing a new vehicle, may be a decision that is decided over a period of time and may not be an older driver’s priority in extending their safe mobility. Alternatively, another explanation may be inadequate power to detect the associations between particular IVTs and the self-regulatory behaviors due to some technologies being uncommon among the participants. For instance, very few participants (approximately 3.65% of the 2990 participants) in the sample reported having an adaptive headlights system in their vehicle at baseline. Due to the prospective design of the LongROAD Study, vehicle changes by participants are being documented over time and the increased prevalence of IVTs may allow for future assessments of their influence on driving behavior.

### 4.2. Limitations

In a review of the larger LongROAD study methodology, Li and colleagues [24] indicated that the present sample may not be representative of the general population of older adults. The older adults in this study are well-educated, have a high annual household income and do not represent ethnic or racial diversity well. The participants may be healthier than the typical population as well. As functional declines are thought to influence self-regulatory behaviors, the sample may be engaging in self-regulatory behaviors less frequently than the general population of older adults. At the time baseline data were collected, it was not known for how long the participants had owned their car. This limitation made it difficult to determine how much time participants may have had to get acquainted with a particular IVT. The present study used the baseline data of an ongoing cohort study. Future studies may explore the relationship of an IVT and self-regulatory behaviors longitudinally.

## 5. Conclusions

This study investigated the role of IVTs with self-regulation and found supporting evidence that there may be an association between the two. Future work in this field should investigate the reasons for this association and explore whether the presence of these IVTs leads to the modification of one’s self-regulatory behavior. Future work should attempt to describe a causal pathway that explains how these factors may lead to self-regulation and ultimately driving cessation. Furthermore, supporting evidence that gender, age, race/ethnicity, income, and social support are associated with self-regulatory driving behaviors is found in this study.

This study provides a glimpse of how technologies could affect older driver behaviors behind the wheel. Such behaviors could ultimately impact their safe mobility as they age. This study examines only four possible self-regulatory driving behaviors. The range of self-regulatory behaviors is extensive. The relationships between driver technologies and such behaviors deserve exploration.

## Figures and Tables

**Table 1:** Demographic Characteristics of the Study Participants (n = 2990).

	n (%)
Age in Years	
65–69	1243 (41.57%)
70–74	1037 (34.68%)
75–79	710 (23.75%)
Income	
Less than USD 20,000	134 (4.48%)
USD 20,000 to USD 49,999	641 (21.44%)
USD 50,000 to USD 79,999	719 (24.05%)
USD 80,000 to USD 99,999	431 (14.41%)
More than USD 100,000	959 (32.07%)
Race	
White, non-Hispanic	2557 (85.52%)
Black, non-Hispanic	212 (7.09%)
Hispanic	81 (2.71%)
Asian, non-Hispanic	64 (2.14%)
Other	76 (2.54%)
Gender	
Male	1404 (46.96%)
Female	1586 (53.04%)
Site	
Denver, Colorado	600 (20.07%)
Cooperstown, New York	601 (20.10%)
Baltimore, Maryland	588 (19.67%)
Ann Arbor, Michigan	601 (20.10%)
San Diego, California	600 (20.07%)

**Table 2:** Description of In-Vehicle Technologies.

Technology	Description
Lane Departure Warning (LDW)	Lane departure warning system can detect the vehicle’s position in a lane and alerts the driver if the vehicle drifts out of the lane.
Forward Collision Warning (FCW)	Forward collision warning system can provide a warning when the vehicle is about to collide with an object, using sensors that could detect objects in front of the vehicle. In some cases, the system can apply the brake to avoid collision.
Blind Spot Warning (BSW)	Blind spot warning systems provide a warning to the driver using sensors that can detect an object to the left or right of the vehicle.
Adaptive Cruise Control (ACC)	Adaptive cruise control adjusts the vehicle speed automatically and maintains a constant headway between the vehicle the vehicle ahead.
Navigation Assistance (NA)	Navigation systems assists the driver to get their destination by providing an on-screen map and turn-by-turn navigation.
Integrated Bluetooth (IB)	Integrated Bluetooth mobile phone systems allow a driver to automatically connect their phones with their vehicles. This allows the driver to make and receive phone calls using the vehicle’s speakers and dashboard interface without having to hold their phones.
Voice Control (VC)	Voice control technologies allow a driver to operate vehicle systems such as the radio and navigation systems using voice commands
Adaptive Headlights (AH)	Adaptive or active headlights can automatically change the direction of the light beam coming from the headlights when the vehicle steers from left to right.

**Table 3:** Proportion of Older Drivers Who Engage in Self-Regulatory Behavior, by In-Vehicle Technology Presence.

	Does not Avoid Behavior	Avoids Behavior Due to Self-Regulation
	% (n)	% (n)
Driving while talking on a mobile phone		
Total Sample (n = 2990)	23.5 (702)	53.3 (1594)
LDW Present (n = 169)	41.4 (70)	39.6 (67)
FCW Present (n = 206)	40.3 (83)	41.8 (86)
BSW Present (n = 303)	38.3 (116)	42.9 (130)
ACC Present (n = 180)	40.0 (72)	41.1 (74)
NA Present (n = 832)	37.9 (315)	42.4 (353)
IB/VC Present (n = 1438)	35.1 (504)	45.5 (654)
AH Present (n = 109)	43.1 (47)	33.9 (37)
Driving at night		
Total Sample (n = 2990)	62.8 (1878)	33.4 (999)
LDW Present (n = 169)	66.3 (112)	29.0 (49)
FCW Present (n = 206)	69.9 (144)	25.7 (53)
BSW Present (n = 303)	65.7 (199)	30.4 (92)
ACC Present (n = 180)	67.2 (121)	27.8 (50)
NA Present (n = 832)	69.2 (576)	27.5 (229)
IB/VC Present (n = 1438)	66.6 (957)	29.9 (430)
AH Present (n = 109)	72.5 (79)	22.9 (25)
Making a left turn when there is no left turn arrow		
Total Sample (n = 2990)	86.0 (2572)	10.4 (311)
LDW Present (n = 169)	86.4 (146)	11.8 (20)
FCW Present (n = 206)	89.3 (184)	9.7 (20)
BSW Present (n = 303)	88.5 (268)	9.2 (28)
ACC Present (n = 180)	89.4 (161)	8.3 (15)
NA Present (n = 832)	89.1 (741)	7.8 (65)
IB/VC Present (n = 1438)	87.4 (1257)	8.8 (127)
AH Present (n = 109)	89.9 (98)	6.4 (7)
Driving in bad weather		
Total Sample (n = 2990)	47.1 (1409)	39.3 (1174)
LDW Present (n = 169)	51.5 (87)	35.5 (60)
FCW Present (n = 206)	53.9 (111)	33.0 (68)
BSW Present (n = 303)	44.6 (135)	38.0 (115)
ACC Present (n = 180)	54.4 (98)	30.0 (54)
NA Present (n = 332)	50.8 (423)	35.1 (292)
IB/VC Present (n = 1438)	49.9 (718)	35.8 (515)
AH Present (n = 109)	46.8 (51)	30.3 (33)

*Note:* Self-regulation is the avoidance of a behavior/situation in response to declining ability. Proportions are displayed across rows. Total samples are on the leftmost column. No avoidance and avoidance due to self-regulation proportions are displayed on the right two columns. LDW = Lane Departure Warning. FCW = Forward Collision Warning. BSW = Blind Spot Warning. ACC = Adaptive Cruise Control. NA = Navigation Assistance. IB/VC = Integrated Bluetooth/Voice Control. AH = Adaptive Headlights.

**Table 4:** Unadjusted Odds Ratios (ORs) and 95% Confidence Intervals (CIs) of Self-Regulatory Behaviors with Respect to In-Vehicle Technology Presence.

	Self-Regulatory Behaviors			
Technology	Avoiding Talking on a Mobile Phone	Avoiding Driving in Bad Weather	Avoiding Driving at Night	Avoiding Left Turns When There Is No Left Arrow
	OR(95% CI)	OR(95% CI)	OR(95% CI)	OR(95% CI)
LDW	0.4(0.3, 0.6) *	-	0.8(0.6, 1.2)	-
FCW	0.4(0.3, 0.6) *	-	0.7(0.5, 0.9) *	-
BSW	0.4(0.3, 0.6) *	-	0.9(0.7, 1.1)	-
ACC	0.4(0.3, 0.6) *	-	0.8(0.5, 1.1)	-
NA	0.4(0.3, 0.4) *	0.8(0.6, 0.9) *	0.7(0.6, 0.8) *	0.7(0.5, 0.9) *
IB/VC	0.3(0.2, 0.3) *	-	-	-
AH	-	0.8(0.5, 1.2)	0.6(0.4, 0.9) *	-

*Note*. IVTs not hypothesized to be related to a particular self-regulatory behavior were not included in the analysis (denoted by dash line). LDW = Lane Departure Warning. FCW = Forward Collision Warning. BSW = Blind Spot Warning. ACC = Adaptive Cruise Control. NA = Navigation Assistance. IB/VC = Integrated Bluetooth/Voice Control. AH = Adaptive Headlights. * *p* < 0.05.

**Table 5:** Adjusted Odds Ratios (ORs) and 95% Confidence Intervals (CIs) from Hierarchical Logistic Regressions Exploring Relationship of In-Vehicle Technologies and Self-Regulation (Block 1).

	Self-Regulatory Behaviors			
	Avoids Talking on a Mobile Phone	Avoids Driving in Bad Weather	Avoids Driving at Night	Avoids Left Turns When There is No Left Arrow
	OR (95% CI)	OR (95% CI)	OR (95% CI)	OR (95% CI)
Block 1	(n = 2176)	(n = 2450)	(n = 2727)	(n = 2735)
Age in Years (Ref: 65–69)				
70–74	1.3 (1.1, 1.7) *	0.9 (0.8, 1.1)	1.0 (0.8, 1.2)	1.2 (0.9, 1.6)
75–79	2.3 (1.7, 3.0) *	1.1 (0.9, 1.4)	1.3 (1.1, 1.7) *	1.8 (1.4, 2.4) *
Income (Ref: < USD 20,000)				
USD 20,000 to USD 49,999	0.7 (0.4, 1.3)	0.6 (0.4, 1.0) *	0.8 (0.6, 1.3)	0.9 (0.5, 1.6)
USD 50,000 to USD 79,999	0.7 (0.4, 1.2)	0.7 (0.4, 1.0)	0.7 (0.4, 1.0)	1.0 (0.5, 1.7)
USD 80,000 to USD 99,999	0.6 (0.3, 1.1)	0.7 (0.5, 1.2)	0.7 (0.5, 1.1)	0.9 (0.5, 1.7)
USD 100,000 or more	0.5 (0.3, 1.0) *	0.6 (0.4, 0.9) *	0.4 (0.3, 0.6) *	0.8 (0.4, 1.4)
Race (Ref: White, non-Hispanic)				
Black, non-Hispanic	0.7 (0.5, 1.0)	1.2 (0.8, 1.7)	2.1 (1.5, 3.0) *	2.0 (1.3, 3.1) *
Hispanic	0.4 (0.2, 0.8) *	0.9 (0.5, 1.5)	1.0 (0.6, 1.7)	1.6 (0.8, 3.2)
Asian, non-Hispanic	1.3 (0.6, 2.8)	2.6 (1.4, 4.8) *	2.5 (1.4, 4.4) *	2.8 (1.4, 5.7) *
Other	0.8 (0.4, 1.5)	0.9 (0.5, 1.6)	1.4 (0.8, 2.3)	1.1 (0.5, 2.5)
Gender (Ref: Male)				
Female	1.4 (1.1, 1.7) *	2.5 (2.1, 2.9) *	2.3 (2.0, 2.8) *	1.5 (1.2, 2.0) *
Marital Status (Ref: Married or Living Together)				
Not Married or Living Together	1.3 (0.6, 1.0) *	1.0 (0.8, 1.2)	0.9 (0.8, 1.1)	1.1 (0.6, 2.0)
Other people depend on you for rides (Ref: No)				
Yes	1.3 (1.0, 1.7) *	1.2 (1.0, 1.4)	0.7 (0.5, 1.0) *	1.5 (1.2, 2.0)
Other people can give you rides (Ref: No)				
Yes	1.1 (0.6, 1.7)	0.8 (0.6, 1.2)	0.9 (0.8, 1.1)	1.1 (0.6, 2.0)
Site (Ref: Denver, Colorado)				
Cooperstown, New York	1.3 (0.9, 1.8)	1.1 (0.8, 1.4)	1.1 (0.9, 1.5)	0.7 (0.5, 1.0)
Baltimore, Maryland	1.0 (0.7, 1.3)	1.3 (1.0, 1.6)	0.7 (0.5, 0.9) *	0.7 (0.5, 1.0)
Ann Arbor, Michigan	1.3 (1.0, 1.8)	1.5 (1.2, 2.0) *	0.8 (0.6, 1.0)	0.7 (0.4, 1.0) *
San Diego, California	1.1 (0.8, 1.5)	0.9 (0.7, 1.2)	0.9 (0.7, 1.2)	0.5 (0.3, 0.8) *

*Note*: * *p* < 0.05.

**Table 6:** Adjusted Odds Ratios (ORs) and 95% Confidence Intervals (CIs) from Hierarchical Logistic Regressions Exploring Relationship of IVTs and Self-Regulation (Block 2).

	Self-Regulatory Behaviors			
	Avoids Talking on a Mobile Phone	Avoids Driving in Bad Weather	Avoids Driving at Night	Avoids Left Turns When There is No Left Arrow
	OR (95% CI)	OR (95% CI)	OR (95% CI)	OR (95% CI)
Block 2	(n = 2176)	(n = 2450)	(n = 2727)	(n = 2735)
In-Vehicle Technology				
LDW	0.9 (0.5, 1.7)	-	-	-
FCW	1.1 (0.6, 2.0)	-	0.8 (0.5, 1.3)	-
BSW	0.8 (0.5, 1.2)	-	-	-
ACC	0.8 (0.5, 1.4)	-	-	-
NA	0.6 (0.5, 0.8) *	0.9 (0.7, 1.0)	0.8 (0.6, 1.0) *	0.7 (0.5,1.0)
IB/VC	0.4 (0.3, 0.5) *	-	-	-
AH	-	-	0.9 (0.5, 1.4)	-

*Note*: IVTs not hypothesized to be related to a particular self-regulatory behavior were not included in the analysis (denoted by dash line). LDW = Lane Departure Warning. FCW = Forward Collision Warning. BSW = Blind Spot Warning. ACC = Adaptive Cruise Control. NA = Navigation Assistance. IB/VC = Integrated Bluetooth/Voice Control. AH = Adaptive Headlights. * *p* < 0.05.
